# Characterization of Fecal Microbiota with Clinical Specimen Using Long-Read and Short-Read Sequencing Platform

**DOI:** 10.3390/ijms21197110

**Published:** 2020-09-26

**Authors:** Po-Li Wei, Ching-Sheng Hung, Yi-Wei Kao, Ying-Chin Lin, Cheng-Yang Lee, Tzu-Hao Chang, Ben-Chang Shia, Jung-Chun Lin

**Affiliations:** 1 Division of Colorectal Surgery, Department of Surgery, Taipei Medical University Hospital, Taipei Medical University, Taipei 110, Taiwan; poliwei@tmu.edu.tw; 2 Cancer Research Center, Taipei Medical University Hospital, Taipei Medical University, Taipei 110, Taiwan; 3 Translational Laboratory, Department of Medical Research, Taipei Medical University Hospital, Taipei Medical University, Taipei 110, Taiwan; 4 Department of Surgery, College of Medicine, Taipei Medical University, Taipei 110, Taiwan; 5 Graduate Institute of Cancer Biology and Drug Discovery, Taipei Medical University, Taipei 110, Taiwan; 6 PhD Program in Medical Biotechnology, College of Medical Science and Technology, Taipei Medical University, Taipei 110, Taiwan; oryx@w.tmu.edu.tw; 7 Department of Laboratory Medicine, Wan Fang Hospital, Taipei Medical University, Taipei 116, Taiwan; 8 Graduate Institute of Business Administration, College of Management. Fu Jen Catholic University, New Taipei City 242062, Taiwan; kyw498762030@gmail.com; 9 Department of Family Medicine, School of Medicine, College of Medicine, Taipei Medical University, Taipei 110, Taiwan; greening1990@gmail.com; 10 Department of Family Medicine, Wan Fang Hospital, Taipei Medical University, Taipei 116, Taiwan; 11 Office of Information Technology, Taipei Medical University, Taipei 106, Taiwan; nathanlee@tmu.edu.tw; 12 Graduate Institute of Biomedical Informatics, Taipei Medical University, Taipei 106, Taiwan; kevinchang@tmu.edu.tw; 13 School of Medical Laboratory Science and Biotechnology, College of Medical Science and Technology, Taipei Medical University, Taipei 110, Taiwan; 14 Pulmonary Research Center, Wan Fang Hospital, Taipei Medical University, Taipei 116, Taiwan

**Keywords:** 16S rRNA, gut microbiota, MinION, MiSeq

## Abstract

Accurate and rapid identification of microbiotic communities using 16S ribosomal (r)RNA sequencing is a critical task for expanding medical and clinical applications. Next-generation sequencing (NGS) is widely considered a practical approach for direct application to communities without the need for in vitro culturing. In this report, a comparative evaluation of short-read (Illumina) and long-read (Oxford Nanopore Technologies (ONT)) platforms toward 16S rRNA sequencing with the same batch of total genomic DNA extracted from fecal samples is presented. Different 16S gene regions were amplified, bar-coded, and sequenced using the Illumina MiSeq and ONT MinION sequencers and corresponding kits. Mapping of the sequenced amplicon using MinION to the entire 16S rRNA gene was analyzed with the cloud-based EPI2ME algorithm. V3–V4 reads generated using MiSeq were aligned by applying the CLC genomics workbench. More than 90% of sequenced reads generated using distinct sequencers were accurately classified at the genus or species level. The misclassification of sequenced reads at the species level between the two approaches was less substantial as expected. Taken together, the comparative results demonstrate that MinION sequencing platform coupled with the corresponding algorithm could function as a practicable strategy in classifying bacterial community to the species level.

## 1. Introduction

A growing body of studies has suggested the potential correlation between the gut microbiotic community and the occurrence of chronic diseases, including diabetes mellitus [1], chronic kidney disease [2], and colorectal cancer [3]. Advances in high-throughput sequencing approaches have allowed tremendous progress in deciphering the composition of gut microbiota in fecal sample without a culturing step [4]. The 16S ribosomal (r)RNA gene is about 1500 bp and is composed of nine variable regions referred to as V1–V9, interspaced with highly conserved regions. Sequencing of 16S rRNA was first applied to characterize identified bacteria independent of the phenotype [5]. With advancements of high-throughput sequencing, also referred to as next-generation sequencing (NGS), it is effective at sequencing respective 16S rRNA genes from mixed bacterial communities without the need for a culturing process [6]. Presently, the Illumina sequencer is the most widely applied sequencing platform to achieve vast quantities of accurate data with limited read lengths [7]. Using the Illumina MiSeq sequencer, reads with a maximum length of 300 bp can be generated and sequenced, thus making it capable of covering one or more variable regions which differ between bacteria at the species level [7]. Beyond 16S rRNA sequencing, shotgun metagenomics carried out using an advanced Illumina platform allows taxonomic profiling at the strain level [8]. Nevertheless, shotgun metagenomics is much more expensive than 16S rRNA and not feasible with highly abundant host DNA [8]. The launch of long-read sequencing developed by Oxford Nanopore Technologies (ONT) achieves direct sequencing toward nucleic acids of microorganisms with sequenced reads of more than 2 Mb, which makes conducting portable data analysis practical [9]. This innovation allows sequencing of the entire 16S rRNA gene, which suggests its potential for more-extensive identification of microorganisms [10]. Moreover, polymerase chain reaction (PCR)-free long-read sequences lessen the impacts of high-GC regions and amplification-mediated bios to PCR-based short-read sequencing, which leads to more-accurate identification of microorganisms [11].

Multiple workflows were established for bioinformatic analyses of NGS results. For short-read sequencing generated using the Illumina platform, commercial software, such as the CLC genomics workbench, or customized workflows are readily available for analyzing microbial communities [12]. Nevertheless, no such recommendation is available for long-read results generated with technology from ONT. The cloud-based EPI2ME (https://epi2me.nanoporetech.com) algorithm contains an analytic workflow toward 16S rRNA analysis using long-read data, which is proprietary software developed and provided by ONT. Moreover, the reference database containing 16S rRNA sequences is essential to allow accurate identification of microbiotic community compositions using the aforementioned tools. The SILVA database is considered an actively updated database that contains curated 16S rRNA sequences, and it is most frequently applied by multiple analytical packages, including the CLC genomics workbench [13,14]. A collection of 16S rRNA sequences and taxonomic information also recently became available in the National Center for Biotechnology Information (NCBI), which is applied to long-read data analyses [15].

In this study, both short-read and long-read platforms were employed to sequence 16S rRNA genes on the same batch of total DNAs extracted from clinical fecal samples. Sequenced reads generated by the Illumina MiSeq and ONT MinION sequencers were, respectively, analyzed using commercial software with optimized databases. The results suggest that MinION sequencing platform can be used to accurately identify major operational taxonomic units (OTUs) in gut microbial community down to the species level.

## 2. Results

### 2.1. Short-Read Sequencing Consistently Classifies Taxonomic Profiles of Gut Microbiota Using 16S rRNA Sequences

**Figure 1:**
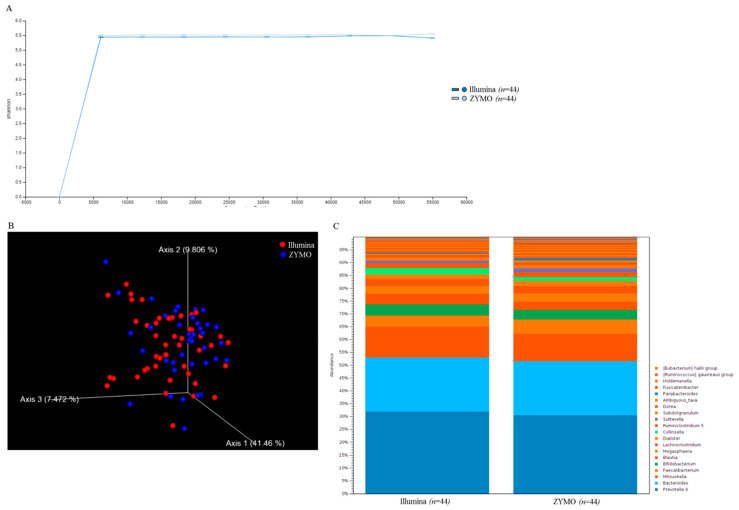
Diversity of taxonomic assignment was consistent between two groups of MiSeq results: (**A**) the α-diversity in two groups of MiSeq data were illustrated using Shannon indices. (**B**) Weighted Unifrac principal component analysis (PCA) was conducted to evaluate the β-diversity indices in two groups of MiSeq data; and (**C**) the relative abundances of top 20 classified OTUs in two groups of MiSeq data are shown in stacked bar chart.

**Figure 2:**
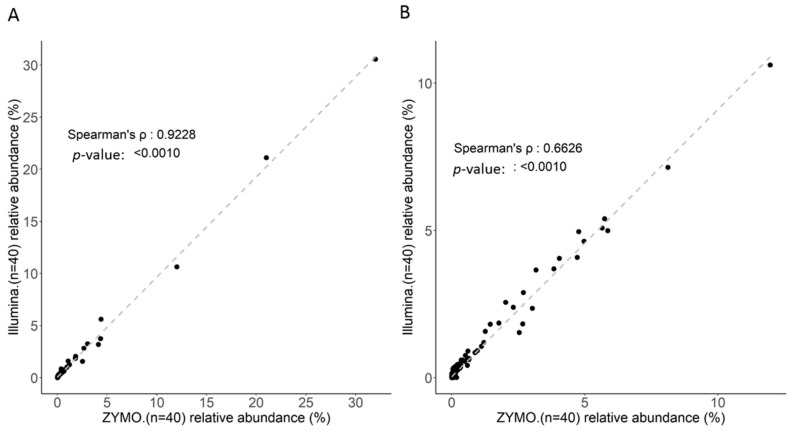
Correlation of identified taxa with two groups of MiSeq data using the Microbial module (CLC) coupled with the SILVA reference for all 44 samples at: (**A**) the genus level; and (**B**) the species level.

The efficiency and consistency of short-read sequencing and corresponding bioinformatics workflow toward the classification of gut microbiota with 16S rRNA sequences were first evaluated. Ten nanograms of total DNA extracted from independent fecal samples (*n* = 44) were subjected to library construction conducted using a distinct protocol (Nextera XT DNA library preparation, Illumina; Quick-16S NGS library Prep lit, Zymo Research). Using the MiSeq sequencer, 221,292 (± 22,944) and 180,386 (± 30,310) reads on average were generated with libraries constructed with distinct kits. The Microbial genomics module (CLC genomics workbench) was employed for synchronous analysis of trimmed and qualified reads with the SILVA reference database down to the generic level. Percentages of correctly classified reads generated using the respective kits for library construction are presented at distinct taxonomic levels (Table 1). In addition to minor variations in the numbers of raw reads, application of distinct protocols toward library construction exhibited similar efficiencies regarding alignment of qualified reads to the SILVA reference from genus to species level (Table 1). No significant variations in alpha-diversity (Figure 1A), beta-diversity (Figure 1B), or identified taxonomic (Figure 1C) profile with distinct libraries were noted in this study. Moreover, the high correlation between the classified taxonomic profiles generated through distinct workflows at the generic level (Figure 2A, *ρ* = 0.9228; *p* < 0.001) or species level was subsequently validated (Figure 2B, *ρ* = 0.6626; *p* < 0.001). Taken together, MiSeq-driven short-read sequencing constituted a consistent and flexible platform for classifying gut microbiota with 16S rRNA sequences with variable experimental workflows.

### 2.2. Long-Read Sequencing is Practicable for Taxonomic Assignment of Microbial Communities

**Figure 3:**
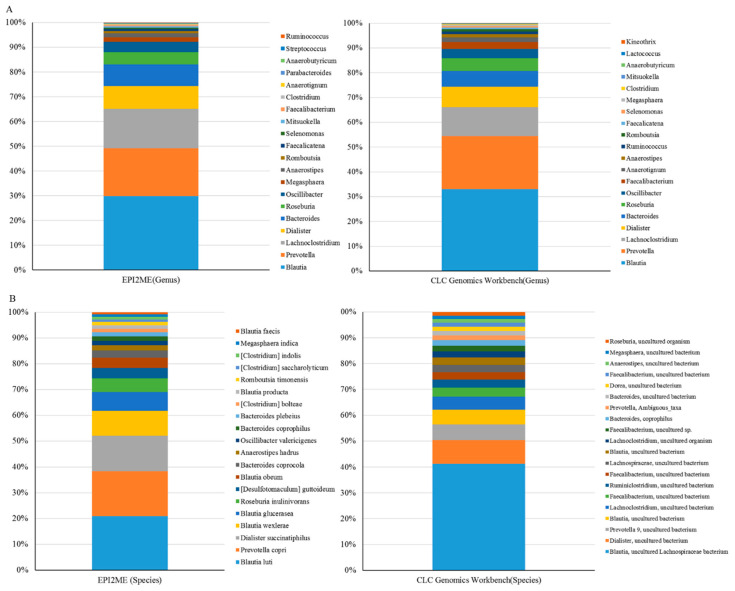
MinION results were subjected to taxonomic assignment using distinct bioinformatics workflows. Long-read amplicons were classified using the EPI2ME algorithm or Microbial module with the SILVA or NCBI 16S databases: at the genus level (**A**); or at the species level (**B**).

**Figure 4:**
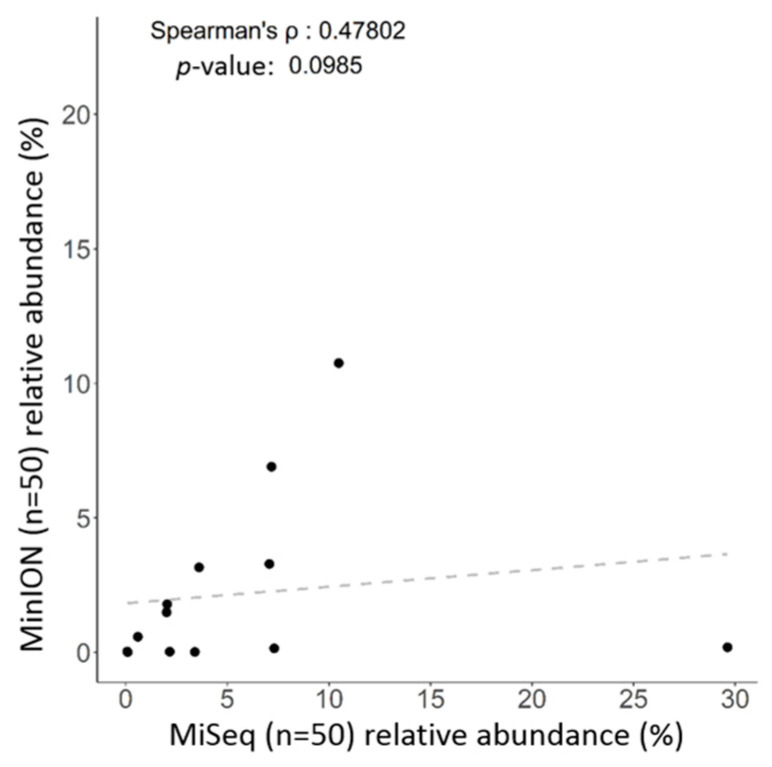
Correlations of identified taxa at the genus level using the Microbial module (CLC) coupled with the SILVA reference or the EPI2ME algorithm with the NCBI database for all 50 samples.

One library was synchronously constructed via amplification of the entire length of the 16S rRNA gene within total DNAs extracted from the same batch of fecal samples (*n* = 50), followed by sequencing with the MinION platform (ONT). The MinKNOW platform was applied for sequencing, and about 60,000 reads were sequenced per sample on average after 17 h. More than 95% of the reads that passed the quality filter were subjected to the following analysis. The Microbial genomics module (CLC) and EPI2ME cloud-based algorithm (ONT) were employed in the bioinformatics analyses with corresponding SILVA and NCBI 16S reference databases. Raw data were split into 10,000 reads to accommodate computational resources with distinct bioinformatic workflows. Application of the EPI2ME with NCBI 16S references allowed classification of the microbial community down to the genus and species levels, whereas use of the Microbial genomics module coupled with the SILVA reference allowed classification of gut microbiota mostly down to the genus level only. An overview regarding read numbers assigned to genus and species levels and unclassified reads using distinct references for the corresponding workflows is shown in Table 2. At the genus level, the majority of mapped reads were correctly classified and assigned to similar taxonomic profiles by using the Microbial genomics module coupled with the SILVA reference (Figure 3A, right bar) or EPI2ME with the NCBI 16S database (Figure 3A, left bar). At the species level, a slight decrease in correctly classified reads with a concomitant increase in the numbers of unclassified reads was noted using both algorithms (Table 2). Nevertheless, the relative levels of correctly assigned reads at the species level using EPI2ME was more significant (Figure 3B, left) compared to the application of Microbial module (Figure 3B, right). A Spearman’s ranking coefficient illustrated variation between bacterial communities analyzed using the Microbial module or the EPI2ME platform at the genus level (Figure 4; *ρ* = 0.478; *p* = 0.0985). Taken together, MinION results coupled with the EPI2ME analysis are practicable for analyzing gut microbial communities.

### 2.3. Correlation between Long Read and Short Read Sequencing toward Taxonomic Assignment of Gut Microbiota

**Figure 5:**
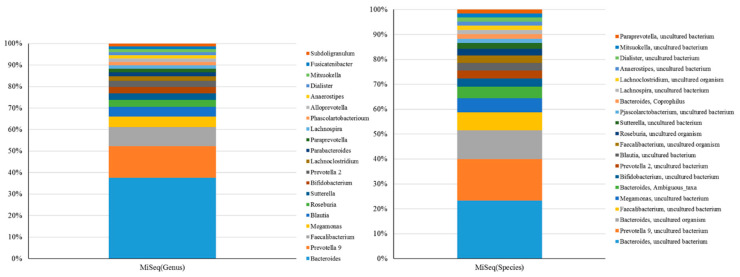
Composition of the 20 most abundant OTUs at genus or species level identified by mapping 16S rRNA gene amplicons sequenced using short-read sequencer against the SILVA reference database.

**Figure 6:**
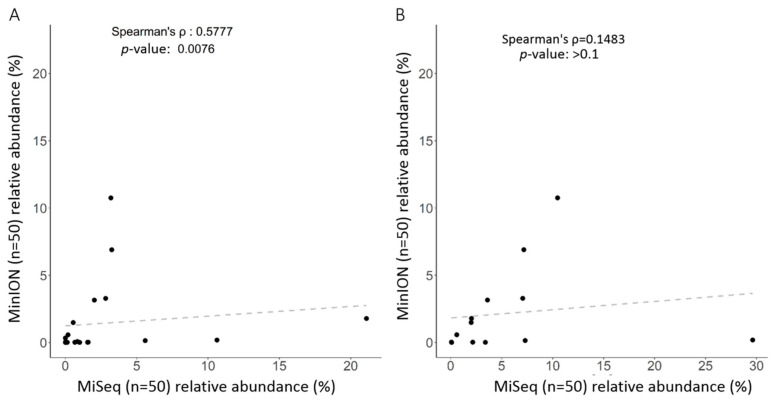
Correlation of identified taxa using short-read sequencing coupled with SILVA reference or long-read sequencing coupled with EPI2ME algorithm with NCBI database: (**A**) at the genus level; or (**B**) at the species level.

Classifications of gut microbiota obtained using the MiSeq or MinION platform were subsequently compared at the genus or species level for 50 independent samples (*n* = 50). Twenty of the most abundant operational taxonomic units (OTUs) at distinct levels are presented in Figure 5. At the genus level, SILVA or NCBI alignment with short-read (Figure 5, left) or long-read amplicons (Figure 3A) showed an insubstantial difference between the taxonomic profiles using the same batch of DNA samples. Only the NCBI alignment with long-read amplicons allowed the classification of the most taxa at the species level (Figure 3B, left), even though several OTUs were synchronously identified when both long-read and short-read amplicons (Figure 5, right) were used. Nevertheless, a Spearman’s ranking coefficient still demonstrated close bacterial communities obtained using the MiSeq and MinION platforms at the genus level (Figure 6, left; *ρ* = 0.578, *p* = 0.0076). Species-level assignment showed substantial differences between the taxonomic profiles with short-read and long-read sequencing coupled with alignment to SILVA and NCBI database (Figure 6, right; *ρ* = 0.1483, *p* > 0.1). These results illustrate the application of long-read amplicons coupled with the EPI2ME algorithm to classify gut microbial communities.

## 3. Discussion

Short-read and long-read sequencing approaches allow rapid, high-throughput, and accurate classification of bacterial communities with identification of 16S rRNA genes [16]. Nevertheless, the methodology of sequencing, the length of sequenced read, and analytic workflow regarding short-read and long-read sequencing is distinct. Recently, both long reads and short reads can be subjected to the same analytic workflow, including Microbial module. Prior to further clinical applications, we compared the performances of short reads (MiSeq; Illumina) and long reads (MinION; ONT) for taxonomic profiling by employing DNA samples prepared from clinical fecal samples and a bacterial reference sample. The sequenced reads were accessed using commercial software to generate the taxonomic profile of each sample, and their correlations were further evaluated.

Oxford Nanopore Technology possesses an advantage of long-read output (over 2.3 Mb according to ONT announcement) over other high-throughput sequencing platforms. The characteristic is practicable for rapid identification of microorganism including bacteria and viruses [17,18,19]. The MinION is recently applied as a rapid and cost-effective sequencing platform that generated long reads to allow sequencing of the entire 16S rRNA gene, which may function as an alternative approach to provide high-resolution results regarding bacterial classification [20]. To evaluate the accuracy of the technology of ONT, the reference standard containing genomic DNAs prepared from eight bacterial and two fungal species were previously subjected to long-read sequencing, followed by two bioinformatics analyses using the CLC genomics workbench or EPI2ME with the SILVA and NCBI 16S reference databases [21]. The high-similarity taxonomy profiles between the reference standard and analytic results generated using distinct workflows suggested the reliability of ONT results for bacterial classification at the specific level. Therefore, application of reference databases was widely considered a critical factor to greatly affect taxonomic classifications [22]. Although differential taxonomy profiles of clinical samples were shown at the genus or species levels using two different bioinformatics analyses in this study, the setting or workflow and employed reference were highly relevant to the identified results of MinION sequencing. For EPI2ME, the employed parameter involved in implementation of the algorithm was absent, which might be considered a “black box” [23,24]. More related research is required to polish the accuracy of error-prone long reads against distinct reference databases.

Nevertheless, the technology of ONT exhibited advantages, including portability, real-time analysis, and time-effectiveness compared to other sequencing platforms or traditional strategies for microbiological studies. Despite the relatively high read error rates of ONT sequencing compared to short-read sequencing, our results illustrate that sequencing of the entire 16S rRNA gene practically improved the accuracy of the classification of the microbial community at the generic and specific levels compared to sequencing of variable regions within the 16S rRNA gene. Consequently, MinION sequencing platform is now applied for classifying differential gut microbial community in distinct clinical specimen in our ongoing study.

## 4. Materials and Methods

### 4.1. Ethics Statement for Use of Clinical Samples

This study was approved by the Joint Institutional Review Board of Taipei Medical University (TMU; approval No. 201901013). Clinical subjects were recruited from the Division of Colorectal Surgery at TMU and the Department of Family Medicine at Wan Fang Hospital, TMU. Exclusion criteria for clinical subjects included use of antibiotics, a history of chemotherapy or radiation therapy, and regular use of a fecal softener within 3 months.

### 4.2. Bacterial DNA Extraction

Fecal samples were collected in DNA/RNA Shield Fecal Collection tubes (Zymo Research, Irvine, CA, USA) and mixed with fixation solution. Total genomic DNAs were extracted from fecal samples using a Quick-DNA Fecal/Soil Microbe Microprep Kit (Zymo Research) according to the manufacturer’s instructions. The quantity and purity of the extracted genomic DNA were evaluated with a fluorometric assay (GeneCopoeia, Rockville, MD, USA) and stored at −80 °C until used.

### 4.3. 16S rRNA Gene Sequencing

For short-read sequencing, 10 ng of genomic DNA samples extracted from a fecal sample were subjected to library construction using the Nextera XT Library Prep Kit (Illumina, CA, USA) or Quick-16S NGS Library Prep Kit (Zymo Research) according the manufacturer’s protocol. Clonal amplification was conducted using the Illumina Miseq platform with the Miseq Reagent kit v3 for 600 cycles. The length of sequenced reads was 2 × 300 nucleotides (nt), and the total read number of individual DNA samples was 50,000–100,000 on average. For long-read sequencing, 10 ng of total genomic DNA were subjected to library construction using the 16S Bar-coding kit (SQK-16S024; ONT, Oxford, UK) according to the manufacturer’s protocol. The bar-coded libraries were loaded and sequenced on MinION flow cells (FLO-MIN106D R9.4.1 using the MinION instrument (ONT). After 17 h, the total read number of individual samples was 60,000–100,000, and the length of sequenced read was 1532 nt in this study.

### 4.4. Bioinformatic Analysis

Short reads generated and sequenced by the MiSeq platform were processed using the CLC Genomics workbench v20.0.4 (CLC bio, Denmark). Qualified and trimmed reads were mapped to 16S rRNA references curated in the SILVA database. Taxonomic Profiling and Find Best Matches with K-mer Spectra (Microbial Genomics Module; CLC genomics workbench) was applied for microbe identification. Throughout analysis of long-read sequencing, EPI2ME (https://epi2me.nanoporetech.com), a cloud-based algorithm including analytical workflow for classification of 16S rRNA with MinION results, was applied in this study. MinION-generated sequencing data were first uploaded by using EPI2ME desktop agent and accessed through a web-interface. Analytical results were generated using EPI2ME to present classification of 16S rRNA based on the NCBI database, containing 18,927 16S rRNA reference sequences. In addition, taxonomic profiling of MinION data was synchronously accessed using the Microbial Genomics Module (CLC genomics workbench) with 16S rRNA references downloaded from the SILVA database (version 128). The required parameter for analyzing *16S rRNA* results using EPI2ME or Microbial Genomics Module is default.

### 4.5. Statistical Analysis

Detailed descriptions regarding short-read or long-read sequencing, including the number of total reads in each run or individual sample, read quality, and depth of coverage obtained by MiSeq and Nanopore sequencing, are presented as the mean ± standard error of the mean (SEM). By using R programming, spearman rank-order correlation coefficients (Rs) were analyzed to show agreement in ranking between all compared pairs. Continuous variables were compared using a one-way or two-way analysis of variance (ANOVA) followed by Tukey’s multiple-comparison post-hoc test.

## Figures and Tables

**Table 1:** Classification results of short-read amplicons with SILVA database for taxonomic assignment at genus or species levels using distinct workflow for library construction.

Workflow for Library Construction		Illumina						ZYMO			
Number of Raw reads (*n* = 44)	Number of classified reads (*n* = 44)	Genus		Species		Number of Raw reads (*n* = 44)	Number of classified reads (*n* = 44)	Genus		Species	
		CC	UC	CC	UC			CC	UC	CC	UC
4,937,768	2,482,744	97.34%	2.66%	63.27%	36.73%	2,269,916	1,014,156	98.02%	1.98%	66.45%	33.55%

Abbreviation: CC, correctly classified; UC, unclassified.

**Table 2:** Classification of MinION results for the taxonomic assignment with different bioinformatics workflows using the NCBI 16S or SILVA databases. The numbers of correctly classified reads at the genus and species levels are presented.

MinION Sequencing		EPI2ME					CLC Genomics Workbench			
Number of Raw reads (*n* = 50)	Number of classified reads (*n* = 50)	Genus		Species			Genus		Species	
		CC	UC	CC	UC		CC	UC	CC	UC
5,033,641	5,027,091	97.21%	2.79%	89.74%	11.26%		96.04%	3.96%	72.15%	27.85%
Assigned OTU		257 (Classified reads > 10)		729 (Classified reads > 10)		Assigned OTU	228 (Classified reads > 10)		52 (Classified reads > 10)	

Abbreviation: CC, correctly classified; UC, unclassified.

## Abbreviations

NGS	Next generation sequencing
ONT	Oxford nanopore technology
